# Retroviruses Hijack Chromatin Loops to Drive Oncogene Expression and Highlight the Chromatin Architecture around Proto-Oncogenic Loci

**DOI:** 10.1371/journal.pone.0120256

**Published:** 2015-03-23

**Authors:** Jillian M. Pattison, Jason B. Wright, Michael D. Cole

**Affiliations:** 1 Department of Genetics, Geisel School of Medicine at Dartmouth, Norris Cotton Cancer Center, Lebanon, New Hampshire, United States of America; 2 Department of Pharmacology and Toxicology, Geisel School of Medicine at Dartmouth, Norris Cotton Cancer Center, Lebanon, New Hampshire, United States of America; Texas A&M University, UNITED STATES

## Abstract

The majority of the genome consists of intergenic and non-coding DNA sequences shown to play a major role in different gene regulatory networks. However, the specific potency of these distal elements as well as how these regions exert function across large genomic distances remains unclear. To address these unresolved issues, we closely examined the chromatin architecture around proto-oncogenic loci in the mouse and human genomes to demonstrate a functional role for chromatin looping in distal gene regulation. Using cell culture models, we show that tumorigenic retroviral integration sites within the mouse genome occur near existing large chromatin loops and that this chromatin architecture is maintained within the human genome as well. Significantly, as mutagenesis screens are not feasible in humans, we demonstrate a way to leverage existing screens in mice to identify disease relevant human enhancers and expose novel disease mechanisms. For instance, we characterize the epigenetic landscape upstream of the human *Cyclin D1* locus to find multiple distal interactions that contribute to the complex *cis*-regulation of this cell cycle gene. Furthermore, we characterize a novel distal interaction upstream of the *Cyclin D1* gene which provides mechanistic evidence for the abundant overexpression of *Cyclin D1* occurring in multiple myeloma cells harboring a pathogenic translocation event. Through use of mapped retroviral integrations and translocation breakpoints, our studies highlight the importance of chromatin looping in oncogene expression, elucidate the epigenetic mechanisms crucial for distal *cis*-regulation, and in one particular instance, explain how a translocation event drives tumorigenesis through upregulation of a proto-oncogene.

## Introduction

Sequencing reports within the last decade have revealed that the majority of the human genome is non-coding sequence. Interestingly, despite previous ideas that non-coding DNA implied non-functional elements, there is a striking amount of recent evidence to show that these non-coding regions play an active role in gene regulation. It is well-established that many of these intergenic regions of DNA are highly conserved sequences among species and contain important *cis*-regulatory elements [1]. Furthermore, studies in both mouse and human have found that the higher order chromatin architecture of the genome facilitates the physical interaction between distal *cis*-regulatory elements and gene promoters [2, 3]. The presence of large chromatin loops thus provides a mechanism by which distal elements contribute to transcriptional regulation and ultimately affect gene expression. Although the chromatin structure of the genome greatly adds to the complexity of gene regulation around many proto-oncogenic loci, understanding the nature of these chromatin loops and the specific roles they play within gene regulatory networks remains a challenge. In this study, we explore the existing chromatin architecture near potent oncogenes in both the mouse and human genomes, and determine a novel role for chromatin looping in the human cancer multiple myeloma. Moreover, after classifying these large chromatin loops, we suggest a new way to identify distal functional elements within the intergenic regions of the genome, through examination of the precise mapping of retroviral integrations.

The mapping of retroviral integration sites within the mouse genome was originally employed as a powerful means to identify oncogenes and oncogene cooperation, particularly in hematopoietic cancers [4–7]. Through large-scale identification of integration sites within the mouse genome and the tracking of the closest gene, key drivers of tumorigenesis were identified and confirmed [5, 6]. The Retrovirus and Transposon tagged Cancer Gene Database (RTCGD) provides a list of documented instances when retroviral insertions have led to lymphomas in mice. A number of these introduced retroviruses integrate in close proximity (within 4 kb from the Transcription Start Site) to oncogene promoters. This consequently drives overexpression of nearby genes through the powerful activity of the retroviral enhancer elements and ultimately results in the development of lymphomas in these particular mice [8]. Interestingly, the majority of integration sites are quite distal to the presumed target gene (up to 230 kb away), and moreover, these distal integration sites cluster at specific distances from the gene [8]. Therefore, careful observation of the RTCGD indicates that these retroviral integrations do not appear to be random and that a large number of these cancer-driving integrations lie in distal intergenic regions of the genome. As the depth of knowledge regarding non-coding regions of the genome is expanding, we have considered annotated retroviral integrations in a new way. Instead of focusing on identifying the genes targeted by frequent integrations, here we set out to understand how the retroviruses are driving tumorigenesis by gaining further insight into higher order chromatin architecture and its contributions to distal gene regulation.

Notably, these retroviral integration sites correspond to common distal chromosomal translocation breakpoints mapped in the human genome around proto-oncogenic loci in B- and T-cell malignancies. This overlap further supports the concept that distal *cis*-regulation is critical for gene expression. For example, the large intergenic region upstream of the human *Cyclin D1* (*CCND1*) locus on 11q13 is a hotspot for chromosomal translocations, particularly in multiple myelomas. Multiple breakpoints have been mapped within this region, from 47 kb to 300 kb centromeric to the *CCND1* TSS in hematologic cancer cells [9–17]. As a result of these translocations that juxtapose the powerful *IGH* locus next to 11q13, CCND1 becomes highly overexpressed [13, 18–21], an unusual event as it is not typically expressed in lymphoid or myeloid cells [22, 23]. Although it has always been presumed that *CCND1* becomes aberrantly controlled by the *IGH* locus, the two loci on the rearranged chromosome remain genomically distal to one another; thus how the *IGH* locus could exert transcriptional activation over such an enormous genomic distance remains elusive. In our study, we provide a mechanism through which the potent *IGH* locus acts on *CCND1* in a translocation context by focusing on the higher order chromatin structures present. In total, our mouse and human studies demonstrate that distal intergenic retroviral integration sites map to functional non-coding elements that are important for cancer pathogenesis. We reveal how utilizing data from a mutagenesis screen in mice can highlight and identify potential oncogenic regulators important in human cancer.

## Materials and Methods

### The Retrovirus and Transposon tagged Cancer Gene Database (RTCGD)

The database is publicly available and was mined using the UCSC genome browser. URLs include http://variation.osu.edu/rtcgd/about_us.html and http://genome.ucsc.edu/.

### Cell lines

The mouse T-cell hybridoma line, 49100.2, was cultured in Dulbecco’s Modified Eagle’s Medium with 10% serum and 1% penicillin-streptomycin under standard conditions. The 49100.2 hybridoma line was produced from the fusion of lymph node cells of B6C2D MHV-68 infected mice with BWZ.36 cells and was a generous gift from EJ Usherwood (Dartmouth College) [24]. The established multiple myeloma cell lines, KMS12 [25], H929 [26], and OPM2 [27] (all generous gifts from WM Kuehl, NCI) [14, 15, 17], were maintained in RPMI (Roswell Park Memorial Institute) 1640 with the same supplements.

### Chromosome conformation capture (3C)

The 3C technique was used to test for distal interactions and chromatin looping occurrences. 3C was performed using established procedures [2, 28]. Briefly, cells were crosslinked with 1% formaldehyde for 10 minutes followed by quenching with glycine at a concentration of 125 mM. Cells were then lysed in 0.2% NP-40 lysis buffer (0.2% NP-40, 10 mM Tris pH 8.0, 10 mM NaCl) and dounced using Pestle B. Digestion with HindIII in NEB Buffer 2 (New England Biolabs) was done overnight at 37°C. Reactions were diluted 1:8 and ligations performed for 4 hours at 16°C. Phenol and chloroform extractions followed by ethanol DNA precipitations were performed to isolate the 3C library which was then analyzed by PCR. Densitometry was performed on ethidium bromide gels to quantitate interaction frequencies. Control fragments were artificially produced from genomic DNA to account for primer efficiencies, and all 3C products were normalized to their respective ligated control fragments with the highest crosslinked frequency set to 1. Two independent libraries were assayed through a minimum of three PCRs.

### Statistical analysis

Graphed values are presented as mean ± standard deviation (S.D.).

## Results

### Chromatin loops connect distal regions of DNA corresponding to common retroviral integration sites

Retroviral integration into the mouse genome causes mainly T and B cell lymphomas through the transcriptional activation of specific oncogenes. At the murine *Ccnd1* locus, there is a cluster of integration sites within 2 kb from the *Ccnd1* promoter, which we will refer to as a proximal cluster. Interestingly, there are also two other distinct clusters of integration sites mapped to the 5’ flanking region of *Ccnd1* that reside 100 kb and 170 kb from the gene itself. These distal integration clusters map within a gene desert, and *Ccnd1* is the only nearby gene with potential oncogenic function. While it has been assumed that *Ccnd1* is the mediator of oncogenic activity for these distal viral integration sites, the mechanism has never been resolved. Given the linear distance between the distal retroviral integration sites and the *Ccnd1* promoter, we explored the possibility that there are chromatin loops present that bring distal sites of retroviral integration clusters into close proximity of oncogene promoters throughout the genome.

**Fig 1:**
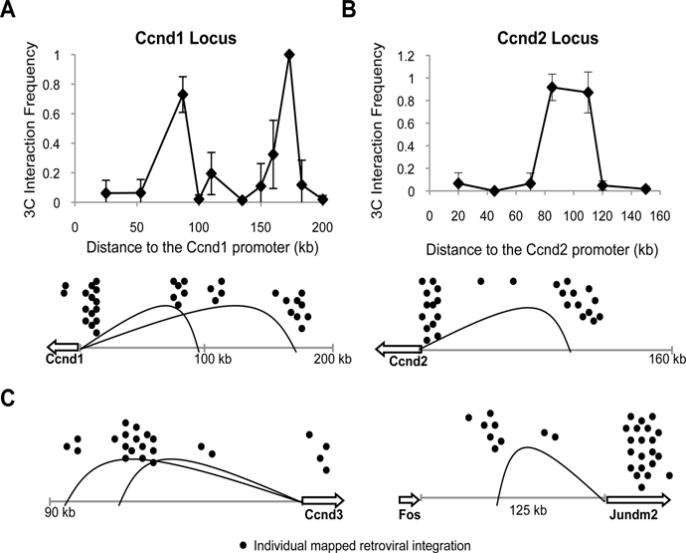
Distal sites of retroviral integration are indicative of chromatin loop presence. A 3C analysis was done in the mouse T-cell hybridoma cell line, 49100.2, and interaction frequency was determined via densitometry of PCR products on an agarose gel stained with ethidium bromide. All interaction frequencies were normalized to corresponding ligation control products and error bars indicate S.D. from triplicate PCRs of two independently generated 3C libraries. Mapped integrations listed in the RTCGD are depicted as black dots. (**a**) Two long-range interactions that correspond to sites of distal integrations are detected at the *Ccnd1* locus. (**b**) One long-range interaction is detected upstream of the *Ccnd2* locus. (**c**) Large chromatin loops are also present at the *Ccnd3* and *Jundm2* loci.

**Fig 2:**
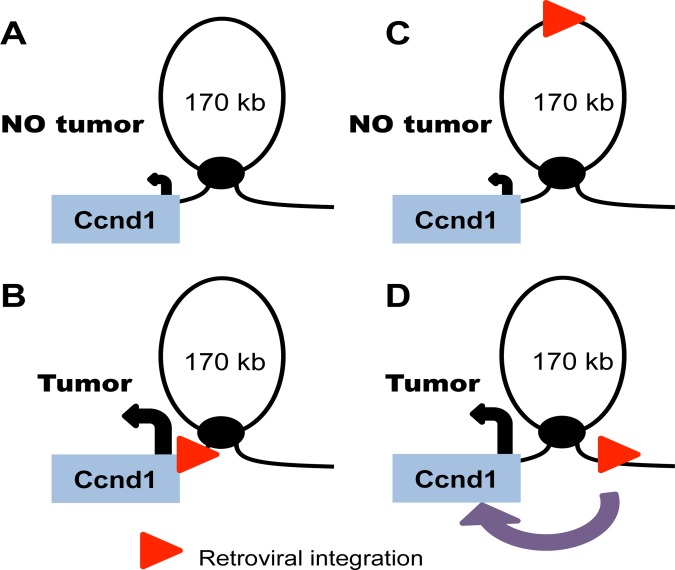
Retroviruses hijack innate chromatin loops to drive oncogene expression. (**a**) A model for the existing chromatin state around *Ccnd1*. (**b**) When a retrovirus integrates proximal to the *Ccnd1* promoter, it drives overexpression through its retroviral enhancer elements and this leads to tumorigenesis. (**c**) When a retrovirus integrates upstream of *Ccnd1* within the chromatin loop, the retroviral enhancers have no physical contact with the *Ccnd1* promoter. This integration has no effect on gene expression and no tumor develops. (**d**) When a retrovirus integrates distal to *Ccnd1* but at the base of a chromatin loop, it is in physical contact with the gene promoter and the enhancer elements can act on this promoter. This scenario leads to tumorigenesis.

There are two modes by which distal gene activation could be facilitated by chromatin architecture. The retroviral LTR could promote looping to distal promoters after integration or, alternately, the retrovirus could hijack preexisting chromatin loops that already juxtapose promoters to distal sites. We decided to test the latter model for several distal retroviral integration clusters and the genes that are assumed to mediate oncogenic activity. To this end, we chose to study a murine T-cell hybridoma cell line, 49100.2, that mimics the cellular context of the lymphomas that develop in mice after retroviral infection [24]. We performed chromosome conformation capture (3C) analysis at different oncogenic loci, and long-range interactions were determined via PCR and verified by sequencing of the PCR products. We found that chromatin loops exist between distal sites of retroviral integration and the promoters of four different proto-oncogenes on four different chromosomes. Two large chromatin loops were detected upstream of *Ccnd1* that link the gene promoter region of DNA 90 kb and 170 kb upstream, regions corresponding to common sites of retroviral integration (Fig. 1A). The *Ccnd2* gene also had one large loop of 100 kb that corresponds to sites of retroviral integration (Fig. 1B). Additional long-range interactions were identified upstream of the proto-oncogenes *Ccnd3* and *Jundm2* (Fig. 1C). Thus these data suggest that retroviruses that integrate distal to oncogenes benefit from the innate chromatin architecture already present as these structures provide selective access to nearby proto-oncogenes. As a result of integration into these genomic regions, retroviruses become physically close to nearby genes and drive misregulation of gene expression and subsequent tumorigenesis (Fig. 2).

### The *Ccnd1* chromatin architecture is conserved between the mouse and human genomes

**Fig 3:**
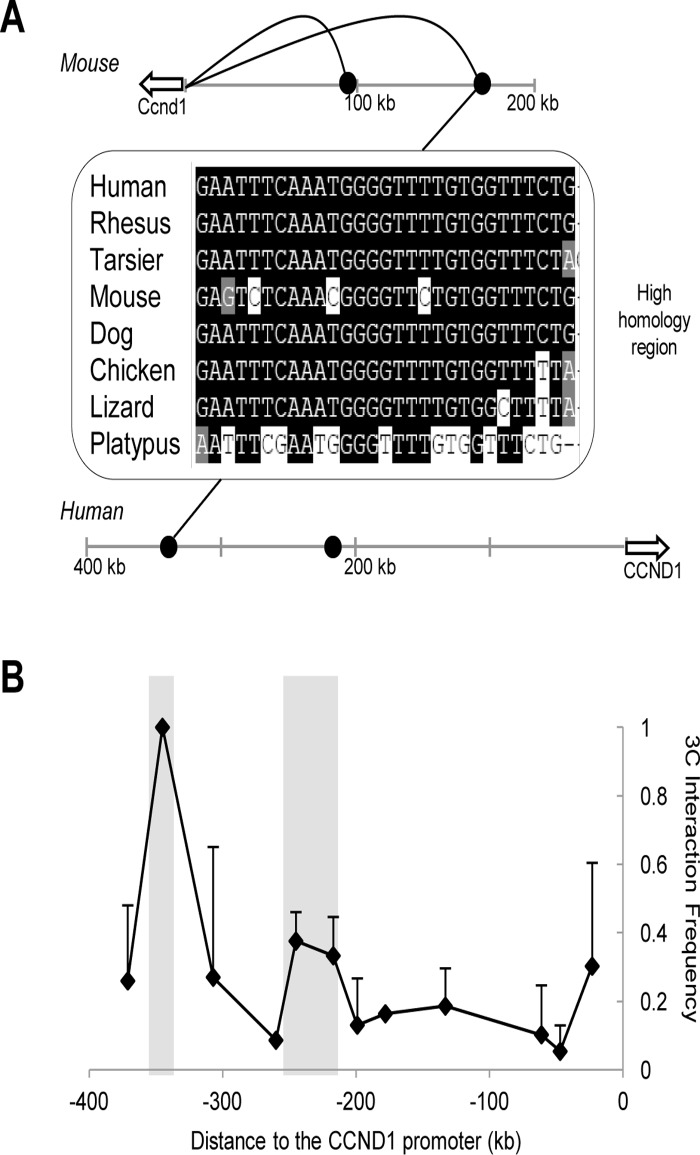
The chromatin architecture of the mouse *Ccnd1* locus is conserved in the human genome. (**a**) The DNA elements at-90 kb and -170 kb, near the bases of the chromatin loops, show high sequence homology throughout evolution. The black curves represent the chromatin loops present at the mouse locus. The sequence depicted is a portion of the high homology region observed at the-170 kb site. A similarly well-conserved homology domain is also observed at the-90 kb region and is represented by the black dots. (**b**) Two chromatin loops, highlighted in the graph, are detected by 3C analysis at the human *CCND1* locus in the multiple myeloma cell line, H929. These loops correspond to the two loops seen within the mouse genome. Two independent 3C libraries were probed through triplicate PCRs and error bars represent S.D.

The finding that chromatin loops link distal retroviral integration clusters into close physical proximity to oncogene promoters led us to more closely examine the regions of intergenic DNA where these common integrations occur. Notably, several DNA elements residing near these chromatin loops are highly conserved sequences marked with specific histone modifications characteristic of enhancers, namely H3K4 dimethylation and H3K27 acetylation (ENCODE ChIP-seq available on http://genome.ucsc.edu/). Given that these intergenic elements are physically linked to proto-oncogene promoters within the murine genome, we hypothesized that the higher order chromatin structures and presumptive enhancers are also conserved within the human genome. We first mapped conserved DNA elements from the murine *Ccnd1* locus to the human genome and found that these regions correspond to unique chromosomal sites in a similar gene desert upstream of the human *CCND1* locus (Fig. 3A). Next we examined whether the large chromatin loops identified in the murine genome also exist at the human locus. We probed the *CCND1* locus in the human H929 multiple myeloma cell line using 3C and subsequently detected two loops at 220 kb and 330 kb in the upstream flanking region (Fig. 3B). Notably, the conserved DNA elements within this upstream region residing at the bases of these loops correspond to the putative enhancers identified in the mouse genome. Thus, it is evident that the higher order architecture of the mouse genome as predicted through common retroviral integration clusters is maintained within the human genome. Interestingly, the sizes of the loops vary between mouse and human but the conservation of specific sequences and enhancer marks intimates that these sites are distal regulatory elements. We suggest that these distal sites are enhancers that may play a key role in the regulation of potent proto-oncogenes.

### Chromosomal translocation induces a large chromatin loop to link *CCND1* with the 3’ immunoglobulin enhancers

**Fig 4:**
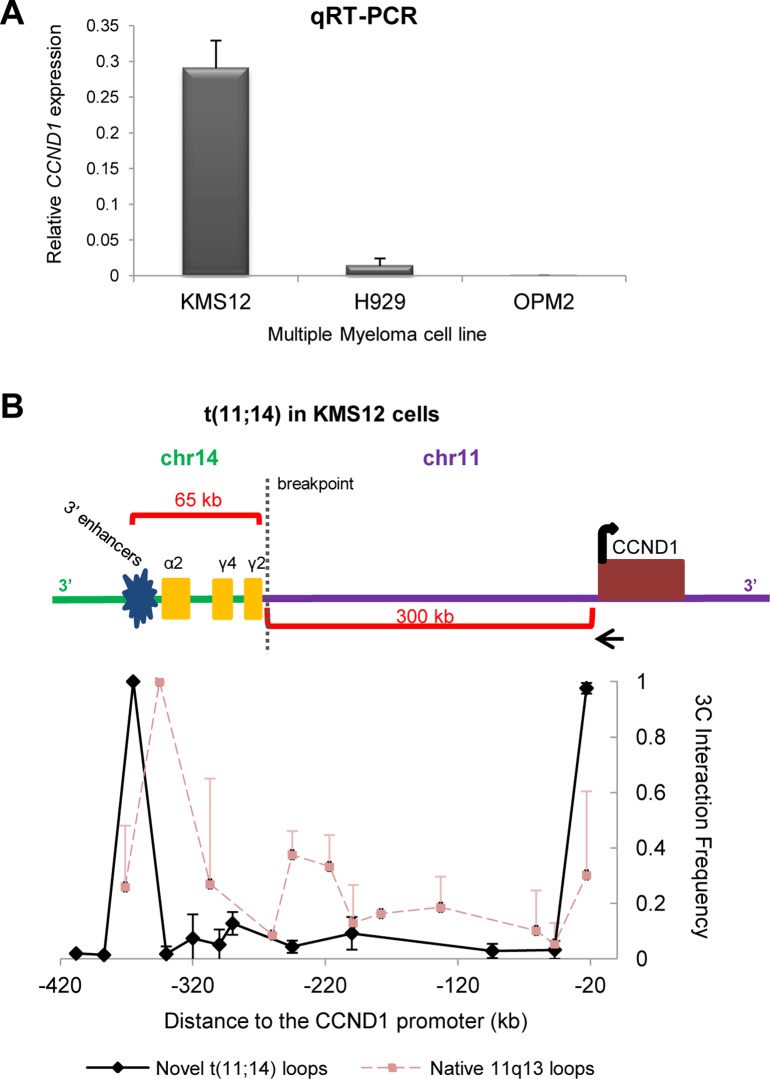
A long-range *cis*-interaction in KMS12 cells connects the *CCND1* promoter and the immunoglobulin 3’ enhancers. (**a**) qRT-PCR analysis showing CCND1 expression relative to Actin in three different multiple myeloma cell lines. KMS12 cells contain a t(11;14) translocation whereas H929 and OPM2 cells have no 11q13 rearrangement. KMS12 cells express high levels of CCND1 as compared to other multiple myeloma cell lines. Expression levels were measured in three independent cell populations and error bars represent S.D. (**b**) A schematic of the translocated chromosome present in KMS12 cells. 3C analysis detects a large chromatin loop that links the *CCND1* promoter with the immunoglobulin 3’ enhancers on the translocated chromosome. The native loops at the *CCND1* locus are no longer detected. The black arrow at the *CCND1* promoter represents the anchor primer. All data were collected from triplicate PCRs of two independent libraries and error bars indicate S.D.

As noted above, the large chromatin loops adjacent to *CCND1* fall within a gene desert devoid of known functional genes. However, the presence of the loops in addition to the high number of retroviral integrations highlights the notion that this non-coding region of DNA plays a critical role in the regulation of *CCND1*. Moreover, to further emphasize its regulatory importance in the human genome, this upstream region is a site of recurrent chromosomal translocation breakpoints, particularly those specific to multiple myelomas [14, 15, 17]. A hallmark of a large fraction of multiple myelomas is a chromosomal translocation t(11:14) in which the *CCND1* locus is joined to the immunoglobulin locus (*IGH* locus). The translocation breakpoints mapped in different cell lines and patients vary within the *IGH* locus on chromosome 14 and extend between 47 kb and 300 kb in the region upstream of *CCND1*. A known consequence of these translocations is overexpression of CCND1 (Fig. 4A) which becomes a driving factor in the development of the myeloma. Although *CCND1* is clearly misregulated in this context, the precise mechanism has never been fully resolved since the translocation breakpoint lies very distal to the gene and gene promoter.

One particular breakpoint in multiple myeloma lies 300 kb upstream of the *CCND1* locus, between the promoter and the base of one of the loops mapped above. We were thus interested to determine if a chromosomal translocation altered the higher order chromatin architecture present in the native *CCND1* locus. To this end, we investigated long-range interactions using 3C analysis in a multiple myeloma cell line, KMS12, that harbors a chromosomal translocation between chromosomes 11 and 14 (Fig. 4B) [29]. The translocation joins *CCND1* to the switch region of the immunoglobulin gamma2 gene (*IGHG2*) in the *IGH* locus, which is 65 kb from the potent enhancers that have been characterized 3’ of the *IGHA2* gene. Numerous probes were designed for the *CCND1* upstream region as well as for sequences within the *IGH* locus. The majority of the probes showed no interaction with the *CCND1* promoter, including those within the gene desert upstream of *CCND1*, which form intrachromosomal loops in other cells. The probes previously used to map the largest loop upstream of *CCND1* are physically separated onto a different chromosome due to the reciprocal translocation, and we found no evidence of any residual interaction that would require an interchromosomal interaction. However, we found a very strong interaction indicative of a chromatin loop between the *CCND1* promoter and the powerful immunoglobulin enhancers located downstream of the translocation breakpoint within the *IGH* locus (Fig. 4B). No interaction between the *CCND1* promoter and the *IGH* enhancers was observed in the absence of chromosomal translocation, as was assessed in the H929 multiple myeloma cells that lack any 11q13 chromosomal rearrangement. Furthermore, we no longer detected the loops of the native *CCND1* locus, presumably as no intact 11q13 chromosomal bands are present in these cells [30]. Thus, the novel interaction that occurs between the 3’ *IGH* enhancers and the *CCND1* promoter appears to surpass any other long-range interactions previously reported in the region flanking *CCND1*. This looping structure thus allows the *IGH* 3’ enhancers to act upon *CCND1* despite the fact that these loci are over 300 kb distant from each other in the linear genome (Fig. 4). This unique chromatin architecture explains the mechanism by which *CCND1* is misregulated in cancers harboring this type of distal chromosomal translocation.

## Discussion

These studies have explored the innate chromatin architecture surrounding multiple proto-oncogenes in relationship to distal clusters of retroviral integration in mouse cancer cells. Our data suggest that when retroviruses integrate near sites of looping, they deregulate gene expression of nearby proto-oncogenes and promote tumorigenesis due to the presence of the innate chromatin architecture. These findings are consistent with a previous report describing chromatin loops and retroviral integration clusters at the murine *Myb* locus [31]. Furthermore, we subsequently were able to translate the architecture of the mouse genome to the human genome. Thus, the use of the retroviral integration database can specifically identify regions of interest within the convoluted, intergenic DNA of the mouse and human genomes to suggest important regulatory regions involved in human cancer.

Interestingly, although it was once believed that retroviral integrations into host genomes were random, multiple studies within the last decade have shed light on the more precise mechanisms of target site selection. It is well-established that retroviruses like MLV integrate just proximal to a gene’s TSS, within DNase-hypersensitive sites, and into regions flanked by patterns of transcription factor binding sites [32–35]. Recent evidence describes the role of BET family proteins in aiding these retroviruses to select active regulatory regions for integration [36]. Significantly, these BET proteins bind acetylated histones, facilitate transcriptional activation, and also directly interact with MLV integrase to guide the retroviruses to areas of active gene transcription [36, 37]. Altogether, it is evident that mapped retroviral integrations provide more than just a resource to identify oncogenes. Instead, these integrations allow for the discovery of potential active regulatory elements throughout intergenic regions of DNA [35]. Our chromatin architecture model described above provides the mechanism through which these distal regulatory sites—where retroviruses are integrating—affect oncogene expression over great genomic distances.

Furthermore, through a more in-depth look at the human *CCND1* locus, it is clear through retroviral integration clusters, chromatin looping, and translocation events that the DNA devoid of genes upstream of *CCND1* is a highly active regulatory region. Recent papers corroborate this finding as enhancer elements are scattered throughout the 5’ region centromeric to *CCND1* [38, 39]. CCND1 plays an important role in the cell cycle and must therefore be tightly regulated in every cell type. Thus it is not surprising that a complicated network of enhancers lies upstream of the gene and that this region of regulatory DNA has been conserved among mammals. Further investigation of these putative enhancer elements will shed light on the complex regulatory system controlling CCND1 expression levels in all cell types.

Additionally, the unique and novel chromatin loop that forms upon a t(11;14) translocation event provides insight into how these large chromosomal rearrangements are leading to deregulation of important oncogenes. Only one other study has demonstrated the formation of a novel long-range *cis*-interaction that occurs upon the joining of two different chromosomes after a translocation event [40]. In a lymphoma bearing a t(14;18) rearrangement, the 3’ *IGH* enhancers are brought into close proximity with the *Bcl2* gene through a large chromatin loop. In turn, overexpression of *Bcl2* occurs within these cancerous cells [40]. This evidence along with the data we present together suggest that there are intrinsic properties within certain regions of the genome, such as the *IGH* locus, *CCND1*, and other proto-oncogenic loci, that allow chromatin loops to instinctively form. When the normal chromatin conformation is disrupted by the presence of a translocation, the intrinsic looping capabilities of distant loci allow for these large chromatin structures to take shape, leading to improper gene regulation of newly-connected genes. Although the translocation breakpoints along chromosome arm 11q13 lie within 47 kb and 300 kb centromeric to *CCND1*, the classified breakpoints characteristic of multiple myelomas are scattered within this upstream region. Thus it is conceived that such a powerful and active genomic region such as the *IGH* locus bulldozes its way into this intergenic space on 11q13 to drive these recurrent translocation events. The randomness of the breakpoints in this intergenic region suggests that there are inherent capabilities and elements of structure within these loci that allow chromatin looping to occur. It is possible that the presence of certain transcription factors, scaffolding proteins, or even non-coding RNAs around these particular loci are responsible for the inherent and dynamic looping capabilities associated with these regions of the genome.

In understanding the chromatin architecture surrounding potent oncogenes and how this higher order structure connects distal regulatory sites, we shed more light on the complex regulation of oncogenes to determine how this regulation goes awry during disease pathogenesis. We propose a novel model whereby the most potent retroviral insertions within the mouse genome promote oncogenesis by benefiting from their integration near preexistent chromatin loops and higher order architecture. These integration sites can therefore be used to predict additional long range chromosomal interactions and to reveal distal gene regulatory elements that control genes involved in growth and oncogenic transformation in humans. This is a powerful tool since mutagenesis screens are not feasible in humans, and thus, by utilizing the data from existing screens, it is possible to identify disease relevant human enhancers and expose novel disease mechanisms. Specifically, through a look into a chromosomal translocation event, we begin to understand the natural inherent looping capabilities of particular loci within the genome. This allows for further comprehension of genome organization and the functional output and significance of this higher order chromatin structure. These two genomic events, namely mapped retroviral integrations and translocation breakpoints, predict the presence and highlight the importance of the chromatin architecture within non-coding regions of DNA that greatly contribute to regulation of proto-oncogenes.
